# Sediment transport capacity of concentrated flows on steep loessial slope with erodible beds

**DOI:** 10.1038/s41598-017-02565-8

**Published:** 2017-05-24

**Authors:** Hai Xiao, Gang Liu, Puling Liu, Fenli Zheng, Jiaqiong Zhang, Feinan Hu

**Affiliations:** 1 State Key Laboratory of Soil Erosion and Dryland Farming on the Loess Plateau, Institute of Soil and Water Conservation, Northwest A&F University, Yangling, 712100 People’s Republic of China; 2 Institute of Soil and Water Conservation of Chinese Academy of Sciences and Ministry of Water Resources, Yangling, 712100 People’s Republic of China

## Abstract

Previous research on sediment transport capacity has been inadequate and incomplete in describing the detachment and transport process of concentrated flows on slope farmlands during rill development. An indoor concentrated flow scouring experiment was carried out on steep loessial soil slope with erodible bed to investigate the sediment transport capacity under different flow rates and slope gradients. The results indicated that the sediment transport capacity increases with increasing flow rate and slope gradient, and these relationships can be described by power functions and exponential functions, respectively. Multivariate, nonlinear regression analysis showed that sediment transport capacity was more sensitive to slope gradient than to flow rate, and it was more sensitive to unit discharge per unit width than to slope gradient for sediment transport capacity in this study. When similar soil was used, the results were similar to those of previous research conducted under both erodible and non-erodible bed conditions. However, the equation derived from previous research under non-erodible bed conditions with for river bed sand tends to overestimate sediment transport capacity in our experiment.

## Introduction

Soil erosion is a key factor for understanding land degradation processes around the world^1–3^. This is especially relevant in areas where soil erosion is very intense due to low infiltration rates and high erodibility^4^. The Loess Plateau is one part of the world where a better understanding of erosion rates and soil erosion processes is necessary^5^. The Loess Plateau in northwest China is susceptible to water erosion and this erosion leads to significant environmental impacts^6^, such as reductions in the quantity and quality of crops^7, 8^. Rill erosion caused by concentrated flow has always been considered a serious problem, as it accounts for approximately 70% of upland erosion in the Loess Plateau^9^.

Sediment transport capacity, defined as the maximum load of sediment that a given flow rate can carry, is essential in many process-based erosion models^10, 11^. It is referred to as net erosion or deposition, respectively, when the actual sediment load is below or above the transport capacity. Thus, it is important to quantify the relationship between detachment rate and sediment load. There have been three different viewpoints for describing the relationship between the soil detachment rate and sediment load: (1) soil detachment is independent of the magnitude of the sediment load; (2) soil detachment is predicted by using probability density functions and (3) soil detachment is modelled as a function of transport capacity deficit^12^. An approach reflecting the third viewpoint proposed by Foster and Meyer^13^ was extensively used, verified and adopted by the famous process-based erosion models, particularly the water erosion prediction project (WEPP) model described in Equation^11, 14, 15^.

1 $$\frac{{D}_{r}}{{D}_{c}}+\frac{{q}_{s}}{{T}_{c}}=1$$

where *D*_*r*_ is the rill detachment rate (kg m^−2^ s^−1^); *D*_*c*_ is the detachment capacity under clear water flow conditions (kg m^−2^ s^−1^); *q*_*s*_ is the sediment load (kg m^−1^ s^−1^) and *T*_*c*_ is the transport capacity (kg m^−1^ s^−1^).

The sediment transport capacity *T*_*c*_ was estimated based on the transport coefficient and the hydraulic shear stress *τ*, which is based on the bed load equation reported by Yalin^16^. In addition to the shear stress *τ*, other hydrodynamic parameters such as stream power and unit stream power have been used to estimate the sediment transport capacity^17, 18^. The sediment transport capacity *T*_*c*_ was closely related with slope gradient and unit discharge per unit width, and empirical formulations were established accordingly^17–20^. Govers^21^ developed a general empirical formula to describe the relationship between sediment transport capacity, slope gradient and unit discharge per unit width as follows:

2 $${T}_{c}=a{S}^{b}{q}^{c}$$

here, *a, b* and *c* are coefficients associated with grain size and with laminar and turbulent-flow regimes, respectively; *S* is the slope gradient (%) and *q* is unit discharge per unit width (m^2^ s^−1^).

Zhang *et al*.^17^ and Wang *et al*.^18^ added river bed sand and native loessial soil to flows to estimate the sediment transport capacity on steep slope with a non-erodible bed (an iron or steel flume bed glued with the test sediment material), respectively. These investigations focused on simulating concentrated flow, but they ignored changes in bed morphology during rill development. In addition, flow hydraulic conditions in a non-erodible bed were different from those in erodible bed during concentrated flow erosion^20^. The well-sorted, non-cohesive sands with different median grain diameters were also used to estimate the sediment transport capacity for soil erosion research under erodible bed conditions by many researchers^21, 22^. However, the physicochemical properties of well-sorted, non-cohesive sands also differ greatly from those of the eroded materials during soil erosion. The eroded materials would change their size and shape during transport^23^, so it is hard to apply these results to soil erosion research directly. Lei *et al*.^24^ assumed that the sediment transport capacity can be obtained when sediment concentration reaches a stable value or when there are no noticeable increases with increasing rill length. Thus, they measured the sediment concentration under different rill lengths and developed a model with a single two-parameter exponential equation. The sediment transport capacity was estimated by multiplying the unit discharge per unit width with the maximum sediment concentration calculated from the single two-parameter exponential equation. However, this experimental method disrupted the continuity of the rill erosion process. Zhang *et al*.^25^ estimated the sediment concentration under different rill lengths without disrupting the continuity of the rill erosion process by using rare earth elements trace method within a 10 cm width rill to quantify the sediment transport capacity in eroding rills. However, this method restricts the development of the rill in the width dimension. The methods mentioned above can evaluate sediment transport capacity under those experiment conditions. Nevertheless, all these methods were inadequate and incomplete in describing the detachment and transport process of concentrated flow on slope farmlands during rill development.

Against background information mentioned above, the purposes of this paper were to: (i) estimate the effects of slope gradient and flow rate on sediment transport capacity on steep loessial soil slope farmlands with erodible beds; (ii) examine the combined effect of slope gradient and flow rate on sediment transport capacity; and (iii) examine the combined effects of slope gradient and unit discharge per unit width on sediment transport capacity.

## Results

### Concentrated flow hydrodynamic characteristics

Table 1 presents the hydrodynamic characteristics of the concentrated flow under a range of different slope gradients and flow rates. All Reynolds numbers (*Re*) were greater than 500, and all Froude numbers (*Fr*) were greater than 1, so the concentrated flows in our research could always be considered to be turbulent rapid super-critical flows. Furthermore, the Reynolds numbers (*Re*) significantly increased when the slope gradient and flow rate were increased (Table 2). Generally, the Froude numbers (*Fr*) decreased as slope gradient and flow rate increased, whereas the Darcy-Weisbach number (*f*) showed the opposite trend (Table 1). No significant relationship was found between the Froude numbers (*Fr*) and slope gradient or between the Darcy-Weisbach number (*f*) and flow rate (Table 2). The Darcy-Weisbach number (*f*) was less than 2.0 under the conditions of 17.6% and 26.8% slopes for all flow rates, but it was much larger than 2.0 under 36.4% and 46.6% slopes for all flow rates except 36.4% slope under a flow rate of 10 L min^−1^.

**Table 1:** Hydrodynamic characteristics of concentrated flow under different slope gradients and flow rates.

Slope gradient (%)	Flow rate (L min^−1^)	Reynolds number (Re)	Froude number (Fr)	Darcy-Weisbach (f)
17.6	10	660.33	2.28	0.48
	15	714.12	2.38	0.31
	20	1917.59	1.24	1.93
	25	2217.03	1.42	1.08
26.8	10	603.51	2.39	0.79
	15	945.22	1.93	1.17
	20	1339.61	1.81	1.20
	25	1778.71	1.71	1.92
36.4	10	697.55	2.98	0.97
	15	1681.45	1.42	3.98
	20	1957.20	1.45	4.45
	25	2661.79	2.07	2.89
46.6	10	1685.93	2.47	3.25
	15	2616.56	1.13	6.96
	20	3453.65	1.12	9.31
	25	4483.75	1.60	5.56

**Table 2:** Pearson correlation coefficients between slope gradient, flow rate and hydrodynamic characteristics.

	Reynolds number (Re)	Froude number (Fr)	Darcy-Weisbach (f)
Slope gradient	0.608*	−0.160	0.800**
Flow rate	0.671**	−0.590*	0.250

*Significant at 0.05 level of probability. **Significant at 0.01 level of probability.

### Effects of slope gradient and flow rate on sediment transport capacity

**Figure 1:**
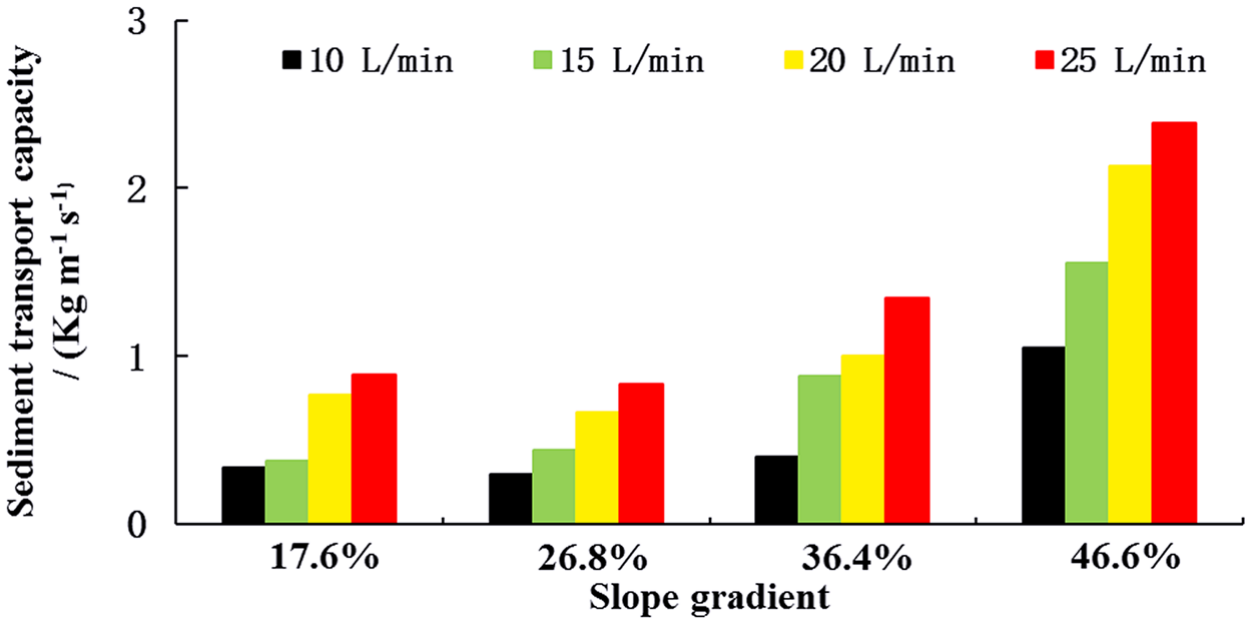
Sediment transport capacity of the concentrated flow under different slope gradients and flow rates.

**Figure 2:**
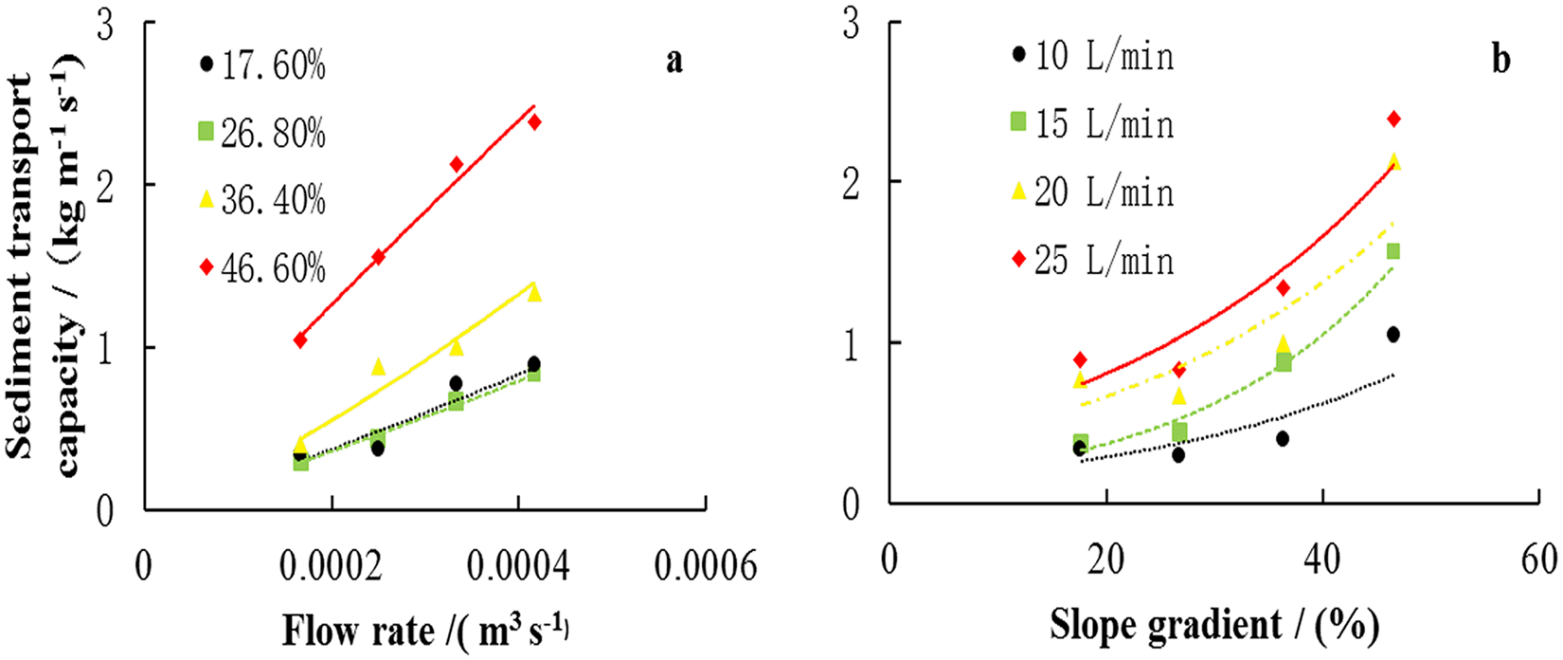
Relationships between sediment transport capacity and: (**a**) flow rate; (**b**) slope gradient.

Sediment transport capacity increased with the increasing flow rate and slope gradient (Fig. 1). For the same level of flow rate, sediment transport capacity values were almost identical on the 17.6% and 26.8% slopes. However, a marked increase across all flow-rates was observed at 36.4% slope, particularly at higher flow rates and this becomes even more pronounced at 46.6% slope (Fig. 2a). Figure 2b shows the increment in sediment transport capacity was small when the slope gradient was less than 36.4%, and it became larger when the slope gradient increased from 36.4% to 46.6% for the same flow rate. A larger increment in sediment transport capacity for a particular certain slope gradient was also observed in previous research^17, 18^. The results in our research showed that the relationships between sediment transport capacity of concentrated flow and flow rate and slope gradient can be described by power functions (R^2^ > 0.859) and exponential functions (R^2^ > 0.714), respectively (Table 3).

**Table 3:** Correlation Coefficients between sediment transport capacity and slope gradient and flow rate.

Slope gradient (%)	Fitting equation	R^2^	Flow rate (L min^−1^)	Fitting equation	R^2^
17.6	y = 7156.111 Q^1.158^	0.859	10	y = 0.133e^0.037S^	0.714
26.8	y = 5862.978 Q^1.138^	0.992	15	y = 0.131e^0.052S^	0.962
36.4	y = 26814.189 Q^1.267^	0.942	20	y = 0.323e^0.036S^	0.768
46.6	y = 3340.450 Q^0.925^	0.989	25	y = 0.394e^0.036S^	0.854

Y is sediment transport capacity (kg m^−1^ s^−1^); Q is flow rate (m^3^ s^−1^); and S is slope gradient (%).

### The combined effects of slope gradient and flow rate on sediment transport capacity

To determine the combined effects of slope gradient and flow rate on sediment transport capacity, a multivariate, nonlinear regression analysis was performed to develop an equation (Eq. 14). The exponents for flow rate and slope gradient were 0.971 and 1.645, respectively, indicating that sediment transport capacity was more sensitive to slope gradient than to flow rate in this study. The correlation coefficient (R^2^) and RMSE derived from Eq. 14 were 0.877 and 0.212, respectively, indicating that Eq. 3 can explain 88.7% of the variance in the sediment transport capacity, with a residual of 0.212.

3 $$\begin{array}{llll}{T}_{c}={\rm{8.143}}{S}^{{\rm{1.645}}}{Q}^{{\rm{0.971}}} & {\rm{n}}=16 & {{\rm{R}}}^{2}=0.887 & {\rm{RMSE}}=0.212\end{array}$$

A multivariate, nonlinear regression analysis was also used to develop an equation (Eq. 4) for confirming the combined effects of slope gradient and unit discharge per unit width on sediment transport capacity. The exponents for unit discharge per unit width and slope gradient were 0.854 and 0.511, respectively, indicating that sediment transport capacity was more sensitive to unit discharge per unit width than to slope gradient in this study. The correlation coefficient (R^2^) and RMSE was 0.978 and 0.094, respectively, suggesting that it can explain 97.8% of the variance in the sediment transport capacity, with a residual of 0.094.

4 $$\begin{array}{llll}{T}_{c}={\rm{42.740}}{S}^{{\rm{0.511}}}{q}^{{\rm{0.854}}} & {\rm{n}}=16 & {R}^{2}=0.978 & {\rm{RMSE}}=0.094\end{array}$$

## Discussion

The exponents of Eq. 4 indicated that the sediment transport capacity was more sensitive to unit discharge per unit width than to slope gradient. This corroborates findings reported by Wang *et al*.^18^. However, the effects degree of slope gradient and unit discharge per unit width on sediment transport capacity were found to be different in different studies. The exponents for unit discharge per unit width and slope gradient obtained by Zhang *et al*.^17^ were 1.237 and 1.227, respectively, indicating that slope gradient and unit discharge per unit width had similar effects on sediment transport capacity. The exponents for unit discharge per unit width and slope gradient in Beasley and Huggins^26^ were 0.5 and 1 when *q* ≤ 0.046 m^2^ s^−1^, but 2 and 1 when *q* > 0.046 m^2^ s^−1^, respectively. Thus, either slope gradient or unit discharge per unit width was likely to be more sensitive to sediment transport capacity depending on the *q* value.

The effect of slope gradient on sediment transport capacity was smaller in Eq. 4 than that in Eq. 3, which can be ascribed to a narrowing concentrated flow on steeper slopes^27^. A narrower concentrated flow can result in a larger value of unit discharge per unit width *q* when flow rate is the same (Eq. 11), leading to a weaker slope gradient effect on sediment transport capacity.

**Figure 3:**
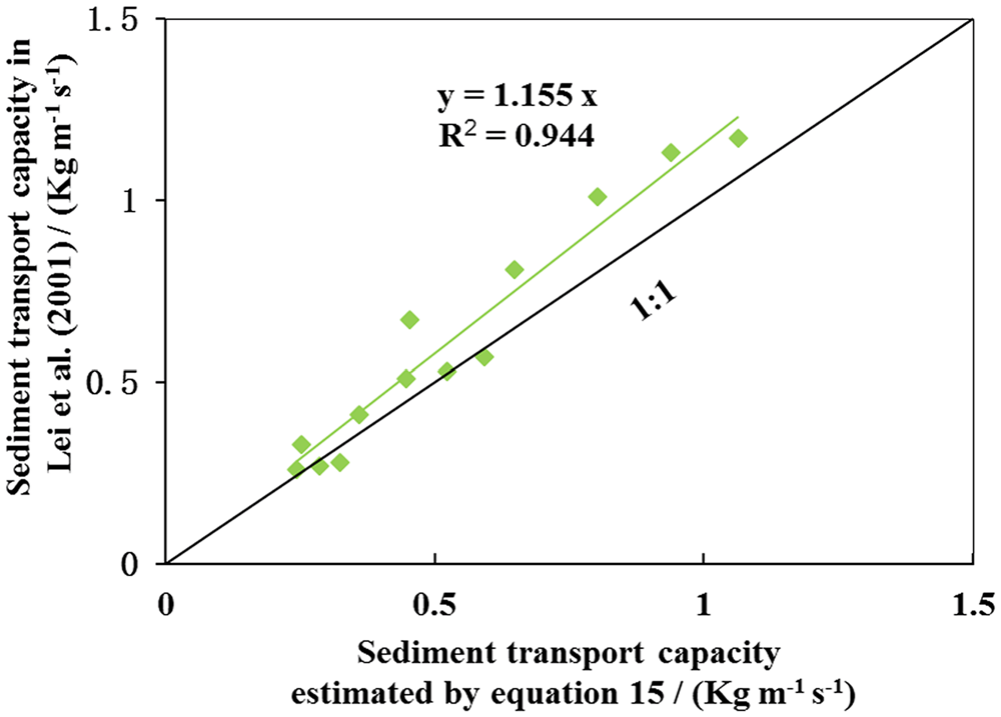
A comparison of sediment transport capacity from Lei *et al*. (2009) under erodible condition and estimated by eq. 4 (*T*_*c*_ = 40.311*S* ^0.551^ *q* ^0.861^, *T*_*c*_ is sediment transport capacity (kg m^−1^ s^−1^), *S* is slope gradient (%) and *q* isdischarge per unit width (m^2^ s^−1^)).

The slope gradient and unit discharge per unit width in Lei *et al*.^24^ were substituted into Eq. 4 to calculate the sediment transport capacity, and the results were compared with the sediment transport capacity values from Lei *et al*.^24^. Two sets of data are depicted in Fig. 3 for further analysis.

All the data were near the 1:1 line, and a linear regression with a zero intercept can describe the relationship between sediment transport capacity in Lei *et al*.^24^ and sediment transport capacity as calculated by Eq. 4 with R^2^ = 0.944. Equation 4 can explain as much as 94.4% of the variance in the sediment transport capacity from Lei *et al*.^24^. The slope of the regression line is 1.155, suggesting that the values of sediment transport capacity from Lei *et al*.^24^ were a slightly larger than those estimated by Eq. 4. Lei *et al*.^24^, measured sediment concentration data were measured separately at different rill lengths. This experimental method disrupted the continuity of the rill erosion process and may have resulted in systematic error. Furthermore, the effect of flow convergence was not taken into account in their experiments, which undoubtedly led to a smaller value of unit discharge per unit width *q*. Thus, their values for sediment transport capacity can be expected to be smaller than the actual values when substituted a smaller *q* is substituted into Eq. 4.

**Figure 4:**
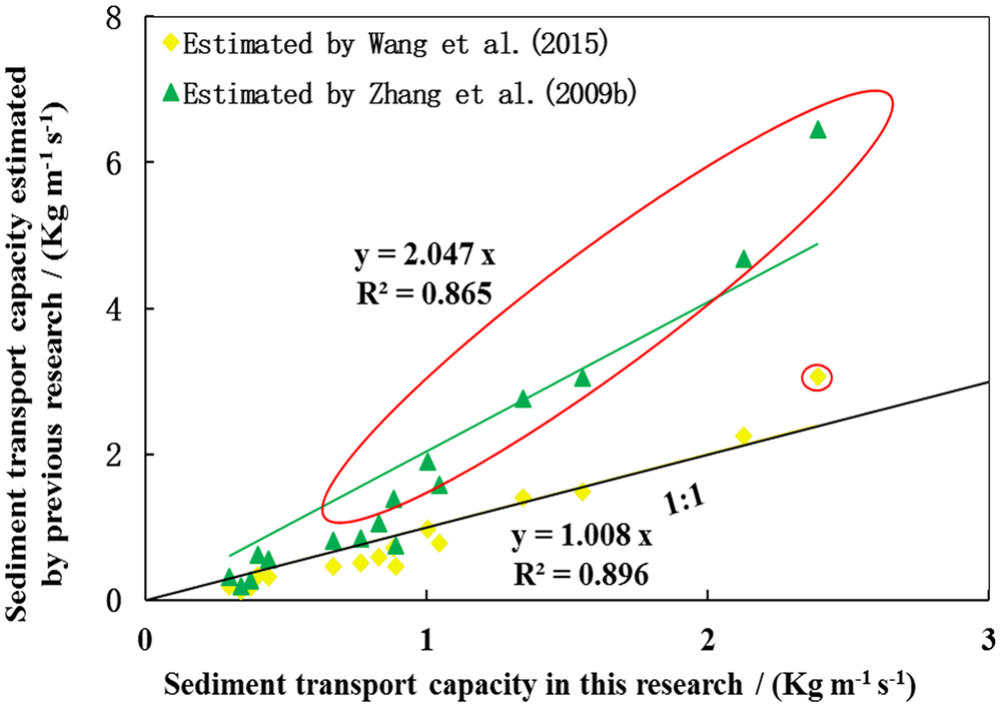
A comparison sediment transport capacity estimated by the equations in Zhang *et al*.^17^ and Wang *et al*.^18^ under non-erodible condition and that in our research.

The equations established by Wang *et al*.^18^ and Zhang *et al.*’s^17^ research were used to calculate the sediment transport capacity, and the results were compared with our data. Three sets of data are depicted in Fig. 4 for further analysis.

The data of sediment transport capacities estimated by Wang *et al*.^18^ and those calculated from our research are near the 1:1 line. A linear regression with zero intercept can properly describe the relationship between them with a R^2^ = 0.896, indicating that the equation established in Wang *et al*.^18^ can explain 89.6% of the variance in the sediment transport capacity in our research. The slope of the regression line is 1.008, suggesting that the values of sediment transport capacity estimated by Wang *et al*.^18^ were slightly higher than those estimated in our research. However, in general, the values of sediment transport capacity estimated by Wang *et al*.^18^ were underestimates at low flow rates and slope gradients and were overestimates at high flow rate and slope gradients. The underestimation may be due to the coarsest fraction of the parent material being deposited while the flow has not yet reached its transporting capacity. The overestimation is probably caused by the lower Darcy-Weisbach number (*f*) under non-erodible bed conditions.

A linear regression with zero intercept can also properly describe the relationship between the sediment transport capacity estimated by Zhang *et al*.^17^ and those calculated from our research with, R^2^ = 0.865. Thus, the equation established in Zhang *et al*.^17^ can explain 86.5% of the variance in sediment transport capacity in our research. The slope of the regression line is 2.047, indicating that the sediment transport capacity estimated by Zhang *et al*.^17^ were much higher than those estimated in our research. The sediment transport capacity estimated by Zhang *et al*.^17^ and those calculated in our research were near the 1:1 line when the sediment transport capacity was small. However, the sediment transport capacity estimated by Zhang *et al*.^17^ was much larger than our values for steep slopes or high unit discharge per unit width (see the circled dots in Fig. 4). Additionally, this phenomenon was observed when comparing sediment transport capacity estimated by Wang *et al*.^17^ and those calculated from our research (see the circled dots in Fig. 4).

The sediment transport capacity under non-erodible bed condition was larger than those in our research for steep slopes or high unit discharge per unit width, probably because of the difference in flow properties. The Darcy-Weisbach number (*f*) under steep slope or high unit discharge per unit width was much larger than under the opposite conditions (Table 1). A larger Darcy-Weisbach number (*f*) indicates much greater resistance to overland flow. Thus a larger proportion of the water’s potential energy is needed to overcome the land surface resistance^28^. The resistance of non-erodible beds is noticeably less than that of erodible beds^20^, resulting in much larger sediment transport capacity in the former.

Our results indicate that Eq. 4 involving slope gradient and unit discharge per unit width, is better than that of Eq. 3, involving slope gradient and flow rate due to its higher R^2^ and lower RMSE (R^2^ = 0.978 > 0.887; RMSE = 0.094 < 0.212). However, the Eq. 3 is easier use than Eq. 4 to estimate the sediment transport capacity in practice because the flow rate data is easier to obtain than unit discharge per unit width.

The equation established in our research can appropriately calculate the sediment transport capacity under the erodible bed conditions and its results are similar to equations established from previous research that used the similar soil. Additionally, when similar soil is used under the non-erodible bed condition, the equations established in previous research can also be used to calculate the sediment transport capacity using our data. However, under non-erodible bed condition and with the river bed sand, the equation established from previous research researches tend to overestimate the sediment transport capacity. Thus, the material used for sediment transport capacity studies in soil erosion research should be the native soil. Additionally, the methods used in previous research under erodible or non-erodible condition were inadequate to model the detachment and transport process of concentrated flow on slope during rill development. In addition, they ignored the narrowing concentrated flow that occurs on steep slope. Those factors would result in an overestimate of sediment transport capacity. Thus, the calculation method used in our research should be recommended when estimating the sediment transport capacity of concentrated flow for soil erosion.

The results of our research were obtained under the conditions of relatively steep slope gradient and low flow rate with a single soil type. The flow hydraulics on the gentle slope gradient were different from those on the steep slope^28^, and soil erosion properties on steep slopes were entirely different from those on gentle slopes^21, 29, 30^. The flow rate or runoff rate has a greater effect on soil erosion mechanisms and sediment transport capacity^26, 31–33^. The sediment particle size also had a great influence on flow hydraulics and the sediment transport process^33, 34^. Thus, larger flow rates and slope gradients, and more types of soil with different particle size distributions, need to be further analyzed.

## Methods

### Materials

Loessial soil, a typical soil in the Loess Plateau collected from Yanan slope farmland (35°21′–37°31′N and 107°41′–110°31′E) in Shaanxi Province, China, was prepared for this research. The mean annual average precipitation and mean daily temperature in the region are 534.4 mm and 9.9 °C, respectively^35^. The low-frequency and high-magnitude rainfall in this plateau continental monsoon climate zone and hilly steep slopes bring a large amount of overland flow and a high rate of soil erosion. Soil was collected from the top 30-cm layer of cultivated land and consisted of 9.1% clay, 59.6% silt and 31.3% sand. It was classified as Calcic Cambisols according to the soil taxonomic system of the United States Department of Agriculture^36^. The bulk density was 1.25 g cm^−3^, and the organic matter content was 1.52 g kg^−1^.

### Experimental design

The soil used in the experiment was air-dried and sieved through a 5 mm mesh. A Malvern Mastersizer 2000 laser diffraction device (Malvern Instruments Ltd., UK) was used to analyse the particle size distribution, and the potassium dichromate oxidation-external heating method was used to analyse the soil organic matter. An indoor concentrated flow scouring experiment was carried out at the State Key Laboratory of Soil Erosion and Dryland Farming on the Loess Plateau in Yangling, China. The experiments were conducted in a slope adjustable plot (5 m length, 0.5 m depth, 1 m width). The plot was set at the designed slope gradient before packing. Packing was performed layer by layer (5 cm depth per layer) to obtain a uniform average bulk density of approximately 1.25 ± 0.03 g cm^−3^ with 40 cm depth. The base of the plot was perforated and covered with a layer of 10 cm sand under the gauze to facilitate even drainage of the percolating water. After packing, the soil was soaked with water until surface flow occurred by using an electric sprayer to further reduce the variability caused by packing.

Sixteen combinations of four flow rates (10, 15, 20, and 25 L min^−1^) and four slope gradients (17.6%, 26.8%, 36.4%, and 46.6%) were tested. Concentrated flow was supplied by a pipe, and the flow rate was adjusted to obtain the desired value (with less than 5% error) outside the plot. The pipe was placed in the middle of the slope surface, 4.75 m away from the outlet of the plot, which guaranteed that the rill could develop freely without being limited in the width dimension and that the concentrated flow would flow smoothly down the slope. The experiment lasted for 10 min following the initiation of runoff. Runoff and sediment were collected in a series of plastic containers at 1 min. intervals over a 10 min. period. The volume of water in each container was measured, and the sediment was dried in an oven and weighed.

Flow depth is difficult to monitor during erosion because of the complicated conditions of flow and bed surface. The flow-width was measured along four sections between 0.5–1.5 m, 1.5–2.5 m, 2.5–3.5 m and 3.5–4.5 m at 1 minute intervals during each 10 minute runoff period by using a ruler. Flow velocity was measured by dye-tracing technique^34^. The mean travel time of the dye tracer moving between the cross sections at the transects 1 m and 4 m away from the outlet of the plot was measured. Flow velocity was calculated as the distance divided by the mean travel time and multiplied by a correction factor of 0.67 for amendment because the measured velocity was larger than the mean flow velocity^37^.

### Equations and data analysis

According to Eq. (1), sediment transport capacity can be estimated using Eq. 5:

5 $${T}_{c}=\frac{{q}_{s}}{(1\,-\,{D}_{r}/{D}_{c})}$$

where *D*_*c*_, the detachment capacity, is the detachment rate under clear flow condition indicating the maximum detachment rate without any other interference.

6 $${D}_{{\rm{c}}}={k}_{r}(\tau -{\tau }_{c})$$

where *k*_*r*_ is rill soil erodibility (s m^−1^); *τ* is the flow shear stress acting on the soil (Pa); *τ*_*c*_ is the critical shear stress of the soil (Pa). The results *k*_*r*_ = 0.55 (s m^−1^) and *τ*_*c*_ = 2.27 (Pa) obtained by using the same soil under clear flow conditions without any interference from Chen^38^ were adopted in Eq. (6) for calculating the detachment capacity.

Shear stress (*τ*, Pa) is defined as the drag force exerted by the flowing water on soil particles per unit bed area^16^.

7 $$\tau =\rho gRJ$$

where *ρ* is the density of water (kg m^−3^); *g* is gravitational acceleration (m s^−2^); *J* (m m^−1^) is the slope gradient; and *R* is the hydraulic radius, which is considered equal to flow depth (*H*) under overland flow conditions (m). The mean flow (*H*, m) depth can be calculated as follow:

8 $$H=\frac{{\rm{Ro}}}{VWt}$$

where *Ro* is runoff during the observation time *t* (m^3^); *V* is flow velocity after amendment, *W* is average flow width during observation time (m); and *t* was observation time (s), *t* = 60. *D*_*r*_, the rill detachment rate (kg m^−2^ s^−1^), is the actually detachment rate during the observation time.

9 $${D}_{r}=\frac{{E}_{r}}{LWt}$$

where *E*_*r*_ is sediment during observation time (kg); *L* is rill length (m), *L* = 4.75. *q*_*s*_, the sediment load (kg m^−1^ s^−1^), is the sediment per width and per time can be estimated by:

10 $${q}_{{\rm{s}}}=\frac{{E}_{r}}{Wt}$$

Unit discharge per unit width, (*q*, m^2^ s^−1^), in Eq. (2) can be estimated using:

11 $$q=\frac{{\rm{Ro}}}{Wt}$$

The average measure *τ* of each event was used to estimate *D*_*c*_ by Eq. (6). The average measure *D*_*r*_ and *q*_*s*_ was estimated using Eqs (9) and (10), respectively. Then those *D*_*c*_, *D*_*r*_, and *q*_*s*_ were substituted into Eq. (5) for estimating sediment transport capacity.

Furthermore, the commonly used hydrodynamics such as the Reynolds number (*Re*), the Froude number (*Fr*), and the Darcy-Weisbach number(*f*) friction coefficient are important parameters that reflect the property of water flow.

The Reynolds number (*Re*), a parameter to reflect the pattern of water flow, is the ratio of inertia forces to viscous forces of the water flow. Based on the theory of open-channel flow dynamics, flow is laminar when Re is less than 500, but turbulent when greater than 500^39^.

12 $${\rm{Re}}=VR/\eta $$

where *η* is the water kinematic viscosity coefficient (m^2^ s^−1^).

The Froude number (*Fr*), a parameter to reflect the regime of water flow, is the ratio of the inertial forces to the gravitational forces of the water flow. Flow is tranquil sub-critical when Fr is less than 1, but rapid super-critical when greater than 1^40^.

13 $${\rm{Fr}}=V/\sqrt{gH}$$

The Darcy-Weisbach number(*f*) friction coefficient is used to characterize the retardation of flow:

14 $${\rm{f}}=8gRJ/{V}^{2}$$

A multivariate, non-linear regression analysis method was applied to develop equations to describe the relations of sediment transport capacity with flow rate and slope, and with unit discharge per unit width and slope as well by SPSS21.0 (IBM). The correlation coefficient (R^2^) and root mean square error (RMSE) were used to evaluate the performance of equations.

15 $$RMSE=\sqrt{\frac{{\sum }_{i=1}^{n}{({X}_{obs,i}-{X}_{\mathrm{mod}el,i})}^{2}}{n}}$$

where *X*_*obs,i*_ is observed values and *X*_*model, i*_ is modelled values.
